# A phase Ib study evaluating the safety and efficacy of IBI310 plus sintilimab in patients with advanced non‐small‐cell lung cancer who have progressed after anti‐PD‐1/L1 therapy

**DOI:** 10.1002/cam4.6855

**Published:** 2024-01-12

**Authors:** Yuguang Zhao, Xiao Chen, Jun Yao, Jianlin Long, Yong Mao, Di Wu, Aimin Zang, Jun Zhao, Ziling Liu, Rui Meng, Ye Chen, Yang Luo, Qun Guo, Li Li, Jiuwei Cui

**Affiliations:** ^1^ The First Hospital of Jilin University Changchun China; ^2^ The First Affiliated Hospital of Henan University of Science and Technology Luoyang China; ^3^ Chongqing University Affiliated Cancer Hospital Chongqing China; ^4^ Affiliated Hospital of Jiangnan University Wuxi China; ^5^ Affiliated Hospital of Hebei University Baoding China; ^6^ Beijing Cancer Hospital Beijing China; ^7^ Union Hospital Affiliated to Tongji Medical College of Huazhong University of Science and Technology Wuhan China; ^8^ Innovent Biologics, Inc. Suzhou China

## Abstract

**Background:**

The development of immune checkpoint inhibitors has made a significant breakthrough in the treatment of non‐small‐cell lung cancer (NSCLC). However, there remains a huge unmet clinical need for patients with acquired resistance after initial treatment response.

**Methods:**

This study evaluated the combination of IBI310 (an anti‐cytotoxic T lymphocyte‐associated antigen‐4 [CTLA‐4] antibody) and sintilimab (an anti‐programmed death 1 [PD‐1]) antibody) in NSCLC patients who have previously been treated with anti‐PD‐1/ligand (L)1 and acquired resistance. The patients were randomly assigned to receive either a lower dose of IBI310 (1 mg/kg Q3W, cohort A) or a higher dose of IBI310 (3 mg/kg Q3W, cohort B) in combination with sintilimab (200 mg Q3W). The primary endpoints of the study were objective response rate (ORR) assessed by RECISTv1.1 and safety, while secondary endpoints included disease control rate (DCR), progression‐free survival (PFS), and overall survival (OS).

**Results:**

As of November 2, 2023, the study had enrolled 30 patients, with 15 patients in each cohort. The ORR was 13.3% (2/15, 95% confidence interval [CI], 1.7–40.5) in cohort B. DCR were 46.7% (95% CI, 21.3–73.4) and 66.7% (95% CI, 38.4–88.2) in cohorts A and B, respectively. In cohorts A and B of this trial, the median follow‐up times were 4.2 and 5.6 months, respectively. Median PFS was 1.45 (95% CI, 1.35–2.73) versus 2.73 (95% CI, 1.41–4.90) months for cohort A versus B; the median OS was 7.03 (95% CI, 3.09–not calculable [NC]) months in cohort A and 8.90 (95% CI, 5.13–NC) months in cohort B. Of the 30 patients, 86.7% in both cohorts experienced treatment‐related adverse events (TRAEs) with Grade ≥3 TRAEs occurring in 40% and 53.3% of patients in cohorts A and B, respectively.

**Conclusion:**

IBI310 3 mg/kg Q3W plus sintilimab was effective in a small number of previously treated anti‐PD‐1/L1‐resistant NSCLC patients.

## 1 INTRODUCTION

There were approximately 2.2 million new cases of lung cancer and 1.8 million deaths caused by the disease in 2020. Lung cancer ranks the second for incidence and is the leading cause of mortality in the world.^1^ For advanced non‐small‐cell lung cancer (NSCLC) that does not carry mutations in the target, the standard treatment had lately evolved away from chemotherapy and toward immunotherapy. Immunotherapy ± chemotherapy has quickly increased the first line treatment choices for patients with advanced NSCLC not carrying targeted mutations in contemporary clinical practices. These immunotherapy‐based regimens included pembrolizumab, atezolizumab, cemiplimab, and dual immune checkpoint inhibitors (nivolumab plus ipilimumab [an anti‐CTLA‐4 antibody] ± chemotherapy), sintilimab plus chemotherapy and pembrolizumab plus chemotherapy, and atezolizumab plus chemotherapy ± bevacizumab (for non‐squamous NSCLC).^2^, ^3^, ^4^, ^5^, ^6^, ^7^, ^8^, ^9^, ^10^ Despite their compelling antitumor activity of these therapies, nearly 70% of patients with advanced NSCLC do not achieve lasting clinical benefit from immune checkpoint inhibitor (ICI)‐based therapies.^11^ Resistance to ICI therapy is a major area of unmet clinical need for patients with NSCLC.

A previous study on the mechanisms of adaptive resistance observed CTLA‐4 expression upregulation of CD8 T cells in two patients with adaptive resistance to anti‐PD‐1 treatment.^12^ Blocking CTLA‐4 has the potential to overcome the PD‐1/L1 resistance.^13^ IBI310 is a recombinant fully human IgG1 anti‐CTLA‐4 antibody with antibody‐dependent cellular cytotoxicity (ADCC). Sintilimab is a recombinant fully human IgG4 anti‐PD‐1 monoclonal antibody with a higher adaptability for human PD‐1 compared to both nivolumab and pembrolizumab,^14^ and has been approved by the regulatory in China, in classical Hodgkin lymphoma (cHL), NSCLC, hepatocellular carcinoma (HCC), esophageal squamous cell carcinoma (ESCC), and gastric/gastroesophageal junction (G/GEJ) adenocarcinoma.^15^ IBI310 in combination with sintilimab were well‐tolerated and showed a preliminary response benefit in advanced melanoma.^16^ IBI310 combined with sintilimab showed promising efficacy and improved survival benefit in previously heavily treated HCC, with antitumor activity signals observed in the patient who failed previous anti‐PD‐1 therapy.^17^

This study aimed to explore the safety and efficacy of IBI310 plus sintilimab for patients with advanced NSCLC who failed previous anti‐PD‐1/L1‐based therapy.

The purpose of this trial was to investigate the safety and efficacy of IBI310 plus sintilimab in patients with advanced NSCLC who had previously failed anti‐PD‐1/L1‐based treatment.

## 2 MATERIALS AND METHODS

### 2.1 Patients

Adults with histologically proven unresectable NSCLC and at least one detectable target lesion per RECIST v1.1 were eligible. Other key eligibility criteria included: progression after anti‐PD‐1/L1 therapy (for patients who received anti‐PD‐1/L1 as neoadjuvant/adjuvant therapy or consolidation therapy after concurrent chemoradiotherapy, disease progression must occur within 6 months after the end of the treatment), ≤2 previous lines of standard treatment (neoadjuvant/adjuvant therapy or consolidation therapy after concurrent chemoradiotherapy treatment with disease progression occurred within 6 months after the end of therapy was considered as first‐line therapy), no pre‐treated with CTLA‐4 inhibitors, Eastern Cooperative Oncology Group (ECOG) performance status (PS) of 0 or 1. Patients with positive driver genes and primary anti‐PD‐1/L1 resistance were excluded. Consensus on definitions of primary and secondary resistance to systemic anti‐PD‐(L)1 monotherapy for solid tumor settings have been well established by SITC Immunotherapy Resistance Taskforce,^18^ but not to ICI combination therapy (dual immunotherapy or ICI combination with other systemic or topical therapies), although ICI combination chemotherapy is now standard of care (SOC) for NSCLC. In this study, primary resistance to anti‐PD‐1/L1 was defined as the best response of anti‐PD‐1/L1 therapy was disease progression, the rest of which was considered as acquired or adaptive or secondary resistance.

### 2.2 Trial design and treatment

This multicenter, open label, phase I study was registered with ClinicalTrials.gov (NCT NCT05118334). Eligible patients were randomly assigned (1:1) to receive either IBI310 (1 mg/kg Q3W) or IBI310 (3 mg/kg Q3W) in combination with sintilimab (200 mg Q3W) (hereinafter cohort A and cohort B, respectively). Patients would receive assigned treatment until progressive disease (PD), intolerable toxicity, withdrawal of informed consent, death, or for up to a maximum of 2 years, whichever occurred first. The primary of the phase 1 study endpoints were objective response rate (ORR) (complete response [CR] or partial response [PR]) assessed by investigator per RECIST v1.1 and safety. The second endpoints included duration of response (DoR), progression‐free survival (PFS) and overall survival (OS).

### 2.3 Assessments

Baseline tumor assessments with cross‐sectional imaging (CT or MRI) were performed by investigator according to RECIST v1.1, then every 6 weeks for the first 48 weeks, and every 9 weeks thereafter until PD, withdrawal of informed consent, death, or study discontinued, whichever occurred first. Safety assessments began with the signing of informed consent form and continued until 30 or 90 days after discontinuation of study drug. Adverse event (AE) severity was rated according to the National Cancer Institute (NCI) Common Toxicity Criteria Version 5.0 (CTCAE 5.0). Treatment‐related AE (TRAEs) to any study drug and immune‐related AE (irAEs) were assessed by investigator. Only serious AE and irAEs related to any study drug were reported subsequently if patients began a new antitumor therapy within 90 days after treatment discontinuation. Survival assessments were done every 90 days after the last safety visit.

### 2.4 Trial oversight

The study protocol received approval from independent ethics committees at each study site and was conducted in accordance with the tenets outlined in the declaration of Helsinki. Prior to enrollment, written informed consent was obtained from all patients.

### 2.5 Statistical analysis

Efficacy endpoints included ORR (confirmed, complete, and partial response) and PFS assessed by investigator per RECIST v1.1 in each cohort. All enrolled patients who received at least one dose of the investigational drug were evaluated for safety. The exact method based on binomial distribution was used to estimate ORR, CR, PR, and disease control rate (DCR) and the 95% confidence interval [CI]. The Kaplan–Meier survival analysis method was used to time‐to‐event analyses (PFS, and OS). A statistically significant difference was defined as *p* value <0.05. Statistical analysis was carried out using Statistical Analysis System statistical software version 9.4 (SAS Institute).

## 3 RESULTS

### 3.1 Patient characteristics

**FIGURE 1:**
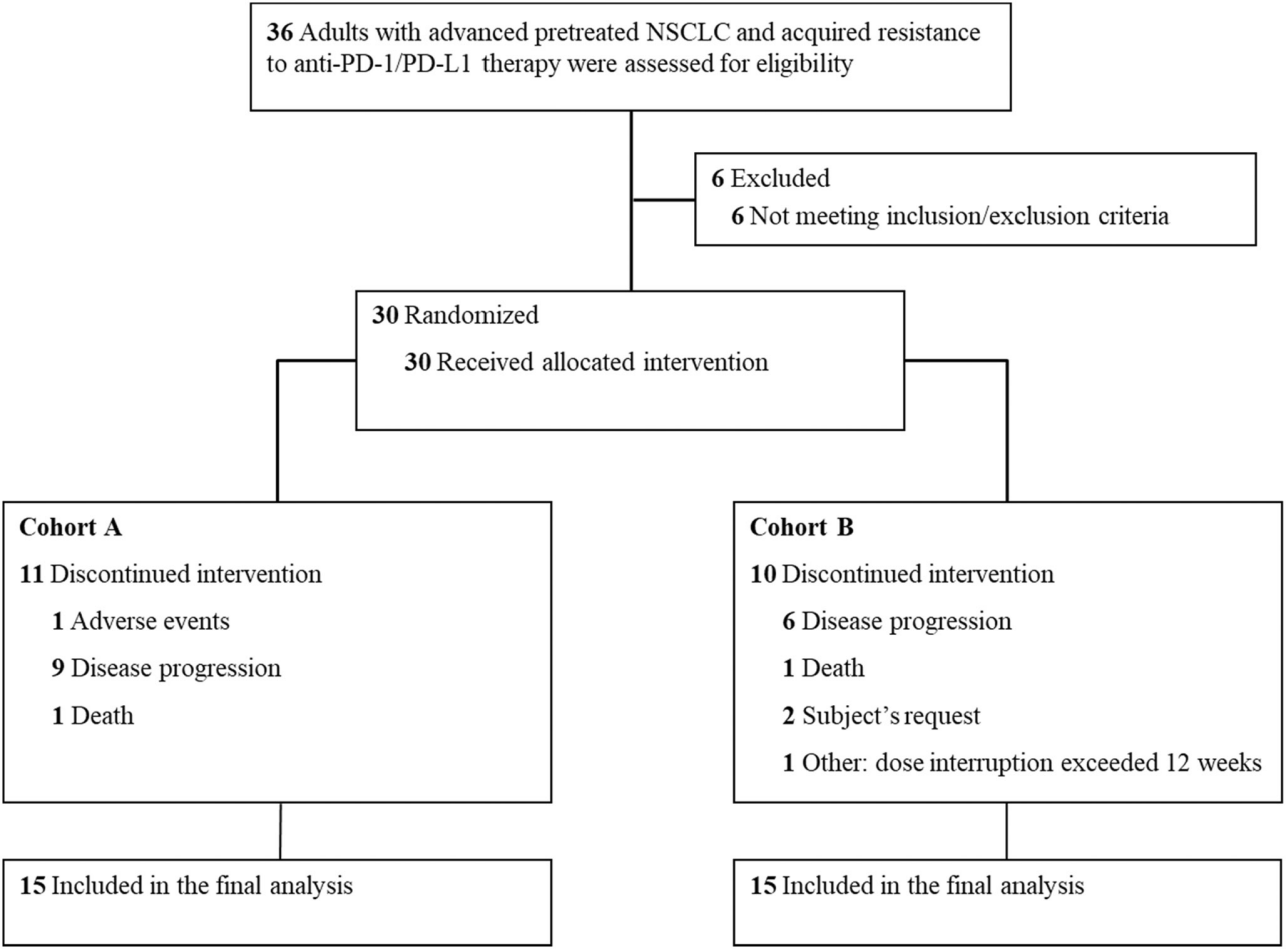
Trial flow chart. Screening, randomization, and flow in the trial of IBI310 (Anti‐CTLA‐4) in combination with sintilimab in patients with advanced non‐small‐cell lung cancer who progressed after anti‐PD‐1/PD‐L1 therapy.

A total of 36 patients were screened from November 18, 2021 through July 29, 2022; 6 of them were excluded because they did not met the eligibility criteria (Figure 1). Finally, 30 patients were eligible for enrollment and randomly assigned (1:1) to cohort A (*n* = 15) or cohort B (*n* = 15). Baseline characteristics of the two cohorts are described in Table 1. In cohort A, median age was 66 years (range 57–74) and 11 (73.3%) patients were men. In cohort B, the median age was 58 years (range 42–72) and 13 (86.7%) patients were men. The main tumor histologic type was adenocarcinoma (8, 53.3%) followed by squamous cell carcinoma (6, 40.0%) in cohort A. However, in cohort B, the main subtype was squamous cell carcinoma (12, 80.0%) followed by adenocarcinoma (3, 20%).

**TABLE 1:** Baseline characteristics.

	Cohort A: IBI310 1 mg/kg plus sintilimab	Cohort B: IBI310 3 mg/kg plus sintilimab	*p* value
	(*n* = 15)	(*n* = 15)	
Age median (range), years	66.0 (57:74)	58.0 (42:72)	0.0237
Male, *n* (%)	11 (73.3)	13 (86.7)	0.6513
ECOG PS, *n* (%)			0.4270
0	3 (20.0)	6 (40.0)	
1	12 (80.0)	9 (60.0)	
Stage, *n* (%)			1.0000
IIIB/IIIC	1 (6.7)	2 (13.3)	
IV	13 (86.6)	12 (80.0)	
Other	1 (6.7)^a^	1 (6.7)^b^	
Tumor histological type, *n* (%)			
Adenocarcinoma	8 (53.3)	3 (20.0)	
Squamous	6 (40.0)	12 (80.0)	
Squamous adenocarcinoma	1 (6.7)	0	
Prior lines of therapy, *n* (%)			0.2723
1	10 (66.7)	6 (40.0)	
2	5 (33.3)	9 (60.0)	
Best response to prior anti‐PD‐ (L)1 therapy, *n* (%)			1.0000
PR	9 (60.0)	8 (53.3)	
SD	6 (40.0)	7 (46.7)	
Last prior line of anti‐PD‐ (L)1 therapy, *n* (%)			1.0000
Yes	14 (93.3)	14 (93.3)	
Prior radiation therapy, *n* (%)			0.0352
Yes	1 (6.7)	7 (46.7)	
CNS metastasis, *n* (%)			1.0000
Yes	1 (6.7)	1 (6.7)	

*Note*: Data are *n* (%) or median (range). *p* values were calculated using *t* test for age and Fisher's exact test for other baseline characteristics.

Abbreviations: CNS, central nervous system; ECOG PS, Eastern Cooperative Oncology Group Performance status.

^a^ T2aN2M0, Stage IIIA‐IIIB.

^b^ TxN2M0, primary tumor cannot be assessed.

Thirty patients received at least one dose of the investigational drug. 11 (73.3.0%, 11/15) and 10 (66.7%, 10/15) patients discontinued treatment in cohorts A and B, respectively. The leading reasons of treatment discontinuation were PD.

### 3.2 Efficacy

**FIGURE 2:**
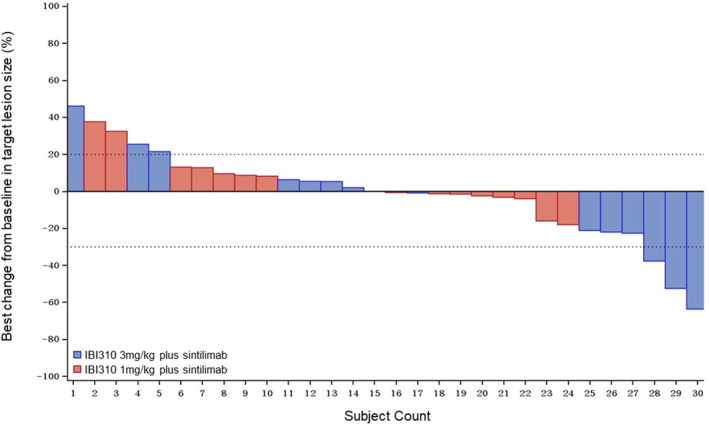
Waterfall plot of response to IBI310 plus sintilimab. Waterfall plot demonstrating best percentage change from baseline in target lesion size.

**FIGURE 3:**
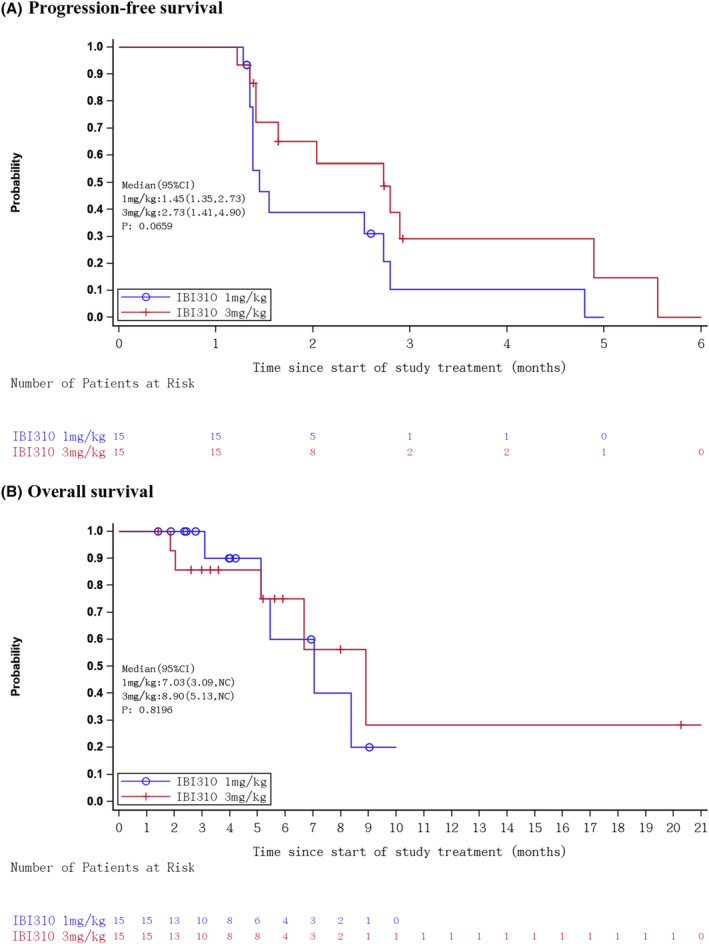
Kaplan–Meier curves of progression‐free survival (PFS) and overall survival (OS) in the acquired PD‐1/PD‐L1 resistant patients. (A) Progression‐free survival and (B) overall survival.

No patients achieved CR or PR in cohort A; two patients achieved PR in cohort B with an ORR of 13.3% (95% CI 1.7–40.5) (Table 2). As of November 2, 2023, the median follow‐up time was 4.2 months (interquartile range [IQR], 2.4–9.0) in cohort A and 5.6 months (IQR, 3.3–8.0) in cohort B. The DoR for the two responders was 2.7 months and 1.6 months. Both responders were adenocarcinoma, whose best response to initial immunotherapy was SD. Seven and 8 patients achieved stable disease in the cohorts A and B, respectively, with DCR of 46.7% (95% CI 21.3–73.4) and 66.7% (95% CI 38.4–88.2). Figure 2 depicts the waterfall plot of best responses in evaluable patients. The median PFS was 1.45 months (95% CI 1.35–2.73) and 2.73 months (95% CI 1.41–4.90), in cohorts A and B, respectively (Figure 3A). Median OS was 7.03 months (95% CI 3.09–not calculable [NC]) in cohort A and 8.90 (95% CI, 5.13–NC) months in cohort B (Figure 3B).

**TABLE 2:** Patient tumor responses.

	Cohort A: IBI310 1 mg/kg plus sintilimab	Cohort B: IBI310 3 mg/kg plus sintilimab	Total
	(*n* = 15)	(*n* = 15)	(*n* = 30)
Partial response	0	2 (13.3)	2 (6.7)
Stable disease	7 (46.7)	8 (53.3)	15 (50.0)
Progressive disease	8 (53.3)	5 (33.3)	13 (43.3)
Objective response rate	0 (0.0, 0.0–21.8)	2 (13.3, 1.7–40.5)	2 (6.7, 0.8–22.1)
Disease control rate	7 (46.7, 21.3–73.4)	10 (66.7, 38.4–88.2)	17 (56.7, 37.4–74.5)

*Note*: Data are *n* (%), *n* (%; 95% CI). Percentages might not sum to 100 because of rounding.

### 3.3 Safety

Safety was assessed in all patients (*n* = 30). Median exposure to study drug was 2.17 months (range, 0.7–7.0) and 1.87 months (range, 0–7.2), in cohorts A and B, respectively. Treatment‐emergent adverse events (TEAEs) occurred in 15 (15/15, 100%) and 14 (14/15, 93.3%) patients in cohorts A and B, respectively. Grade ≥3 TEAEs occurred in 14/15 (93.3%) patients in cohort A and 9/15 (60.0%) in cohort B (Table S1). TEAEs leading to treatment interruption of any treatment drug occurred in 7 (7/15, 46.7%) and 9 (9/15, 60%) patients in cohorts A and B, respectively. One patient experienced TEAEs leading to treatment discontinuation in cohort A while none was observed in cohort B. There were one and two treatment‐related deaths in cohort A (immune‐related hepatitis) and B (one patient died of immune‐related myocarditis and immune‐related hepatitis, another patient died of an unknown cause), respectively.

In cohort A, TRAEs were observed in 86.7% of patients (*n* = 13, 13/15). The main TRAEs were anemia (46.7%), alanine aminotransferase increased (33.3%), aspartate aminotransferase increased (26.7%), and rash (26.7%). Six (40%) patients experienced Grade ≥3 TRAEs. In cohort B, 13 (13/15, 86.7%) patients experienced TRAE, with the most common TRAEs being alanine aminotransferase increased (*n* = 7, 46.7%), hypoalbuminemia (*n* = 6, 40.0%), anemia (*n* = 5, 33.3%), and platelet count decreased (*n* = 5, 33.3%). Grade ≥3 TRAEs were observed in 53.3% of patients (*n* = 8), and 2 patients experienced Grade ≥3 gamma‐glutamyltransferase increased (Table 3).

**TABLE 3:** Adverse events in all patients.

	Cohort A: IBI310 1 mg/kg plus sintilimab(*n* = 15)		Cohort B: IBI310 3 mg/kg plus sintilimab(*n* = 15)	
	Any grade	≥Grade 3	Any grade	≥Grade 3
Any treatment‐related adverse events^a^	13 (86.7)	6 (40.0)	13 (86.7)	8 (53.3)
*p* value for Any grade TRAE	1.0000			
Any‐grade treatment‐related adverse events in 10% or more of treated patients in either group^b^				
Anemia	7 (46.7)	1 (6.7)	5 (33.3)	0
Alanine aminotransferase increased	5 (33.3)	1 (6.7)	7 (46.7)	0
Aspartate aminotransferase increased	4 (26.7)	1 (6.7)	4 (26.7)	1 (6.7)
Rash	4 (26.7)	1 (6.7)	2 (13.3)	1 (6.7)
Blood alkaline phosphatase increased	3 (20.0)	1 (6.7)	4 (26.7)	0
Lipase increased	3 (20.0)	1 (6.7)	3 (20.0)	1 (6.7)
Platelet count decreased	3 (20.0)	1 (6.7)	5 (33.3)	0
Hyponatremia	3 (20.0)	0	3 (20.0)	1 (6.7)
Blood thyroid stimulating hormone increased	3 (20.0)	0	2 (13.3)	0
Gamma‐glutamyltransferase increased	2 (13.3)	0	4 (26.7)	2 (13.3)
Decreased appetite	2 (13.3)	0	3 (20.0)	0
Pyrexia	2 (13.3)	0	3 (20.0)	0
Asthenia	2 (13.3)	0	4 (26.7)	1 (6.7)
Blood bilirubin increased	1 (6.7)	0	3 (20.0)	0
Hypoalbuminemia	1 (6.7)	0	6 (40.0)	0
C‐reactive protein increased	0	0	4 (26.7)	1 (6.7)
Hepatic function abnormal	0	0	3 (20.0)	1 (6.7)
Occult blood positive	0	0	3 (20.0)	0
Hypomagnesaemia	0	0	3 (20.0)	0
Any immune‐related adverse events^c^	9 (60.0)	3 (20.0)	12 (80.0)	6 (40.0)
*p* value for Any grade irAE	0.4270			
Immune‐related adverse events in 10% or more of patients in either group^c^				
Alanine aminotransferase increased	3 (20.0)	1 (6.7)	4 (26.7)	0
Aspartate aminotransferase increased	3 (20.0)	0	4 (26.7)	0
Rash	3 (20.0)	1 (6.7)	2 (13.3)	1 (6.7)
Platelet count decreased	2 (13.3)	0	3 (20.0)	0
Blood thyroid stimulating hormone increased	2 (13.3)	0	1 (6.7)	0
Anemia	2 (13.3)	0	1 (6.7)	0
Adrenal insufficiency	2 (13.3)	0	0	0
Blood alkaline phosphatase increased	1 (6.7)	0	4 (26.7)	0
Gamma‐glutamyltransferase increased	1 (6.7)	0	3 (20.0)	2 (13.3)
Hepatic function abnormal	0	0	3 (20.0)	1 (6.7)
Lipase increased	1 (6.7)	0	3 (20.0)	0
Cortisol decreased	0	0	2 (13.3)	0
Blood bilirubin increased	0	0	2 (13.3)	0
Hyperglycemia	0	0	2 (13.3)	0

*Note*: Data are *n* (%). *p* values were calculated using Fisher's exact test. Treatment‐related adverse event was related to any study drug.

^a^ Adverse events were classified according to Medical Dictionary for Regulatory Activities and graded according to the National Cancer Institute Common Terminology Criteria for Adverse Events, version 5.0. Grading ranges from 1 through 5 (1, mild; 2, moderate; 3, severe; 4, life‐threatening; and 5, death).

^b^ Treatment‐related adverse events to any drug occurring in 10% or more of patients in either group are shown in descending order of frequency in the IBI310 1 mg/kg plus sintilimab group.

^c^ Immune‐related adverse events (AE related to IBI310 or sintilimab) assessed by investigators occurring in 10% or more of patients in either group are listed in descending order of frequency in the IBI310 1 mg/kg plus sintilimab group.

IrAEs occurred in 9 (9/15, 60%) and 12 (12/15, 80%) patients in cohorts A and B, respectively. Most irAEs assessed by investigator were Grade 1 or 2 in severity; Grade 3 or higher events occurred in 3/15 (20.0%) patients in cohort A and 6/15 (40.0%) in cohort B (Table 3). For all 30 patients, the most common irAEs determined by the investigator were alanine aminotransferase increased, and aspartate aminotransferase increased (Table 3). Fourteen (46.7%) patients with irAEs had systemic corticosteroids, most of which were well managed (Table S2).

## 4 DISCUSSION

Numerous efforts have been made in developing novel strategies to overcome ICI resistance across all stages of NSCLC. Different combinations of anti‐PD‐1/L1 antibodies with chemotherapies, radiotherapies, targeted therapies, and other ICIs have been explored in primary or acquired resistance settings, but there remained huge unmet clinical needs.^19^, ^20^, ^21^, ^22^ Molecular mechanisms underlying ICIs response and resistance are complex.^23^ In addition to PD‐1/L1, excessive expression of various alternative immune checkpoints can result in a highly exhausted state of T cells, which can have immunosuppressive effects and hinder the effectiveness of ICI treatment.^12^, ^24^ The development of resistance to ICIs had been observed as a result of the compensatory upregulation of alternative immune checkpoints.^12^, ^24^ Although both CTLA‐4 and PD‐1 are immune checkpoints, their mechanisms of action are different.^25^ CTLA‐4 inhibits CD28‐B7 interactions to modulate T‐cell response induced by antigen‐presenting cells (APCs), whereas PD‐1 is involved in T‐cell receptor (TCR) signaling to regulate effector phase of T‐cell response.^25^ Inhibition of CTLA‐4 primarily impacts the clonal expansion and migration of CD4 T cells, while inhibition of PD‐1/L1 primarily affects the exhausted CD8 T cells.^25^ Therefore, combination of anti‐PD‐1/L1 and anti‐CTLA‐4 treatment could be a rational approach with complementary and synergic effects.^13^

Indeed, dual inhibition of PD‐1/L1 and CTLA‐4 showed improved efficacy than anti‐PD‐1/L1 or anti‐CTLA‐4 monotherapy, chemotherapy, and targeted therapy.^26^ Check‐Mate 227 and Check‐Mate 9LA study demonstrated that combination of nivolumab (anti‐PD‐1) + ipilimumab (anti‐CTLA‐4) as the first line treatment improved survival in NSCLC.^3^, ^5^ In patients who failed previous anti‐PD‐1/L1 treatments, combination of ipilimumab with anti‐PD‐1 antibodies also showed significant antitumor activity.^27^, ^28^ Combination of anti‐PD‐1/L1 and anti‐CTLA‐4 showed similar and manageable toxicity in both ICI‐naïve and ICI‐treated setting in aforementioned studies.

In the present study, we explored sintilimab plus IBI310 in immunotherapy‐resistant NSCLC. Compared with previous studies using anti‐PD‐1 treatment in combination with anti‐CTLA‐4 antibody (e.g., ipilimumab),^3^, ^5^, ^29^, ^30^ no new safety signals were observed in this study. IBI310 3 mg/kg Q3W plus sintilimab 200 mg Q3W seemed less tolerable compared to IBI310 1 mg/kg Q3W plus sintilimab 200 mg Q3W (Table 3), and ipilimumab 1 mg/kg Q6W plus nivolumab 240 mg Q2W as first‐line treatment in NSCLC.^3^ Grade ≥3 irAEs were more common in cohort B than in cohort A (Table 3). A similar dose of anti‐CTLA mAb appeared to exhibit similar safety and toxicity profile in both ICI treated and ICI naïve patients with NSCLC, while a higher dose resulted in increased toxicity. Dual immunotherapy of IBI310 plus sintilimab showed limited efficacy. Only two patients had tumor partial response in cohort B, one of them achieved PR at second tumor assessment at Week 12 but had dose interruption after fourth cycle due to immune‐mediated hepatitis, ending up with PD. High incidence of dose interruption could be a potential factor of the limited efficacy (Table S3). Hence, how to manage toxicity in patients with response is worthy of further exploration. No PR was seen in cohort A with low dose of IBI310 (1 mg/kg) in combination with sintilimab, whereas similar dose of ipilimumab with nivolumab was used in first line treatment of NSCLC, indicating different immune resistance mechanism in upfront and late lines, which needs further investigation.

Some limitations in our study were noted when interpreting the results. The sample size of each treatment arms in our study were relatively small which may reduce the power of the study and increase the margin of error when evaluating treatment efficacy. Biomarkers in our study were also limited. Established biomarkers such as PD‐L1 expression, tumor mutation burden, microsatellite instability, and other emerging markers in tumor immune microenvironment might be informative as well.

In conclusion, the combination of IBI310 and sintilimab demonstrated potential efficacy in a small number of patients who failed previous anti‐PD‐1/L1 treatments. Dose of the two ICIs should be further optimized in future studies to balance toxicity and efficacy.

## AUTHOR CONTRIBUTIONS

**Yuguang Zhao:** Conceptualization (supporting); formal analysis (equal); methodology (equal); validation (equal); writing – original draft (equal); writing – review and editing (equal). **Xiao Chen:** Conceptualization (equal); formal analysis (equal); methodology (equal); validation (equal); writing – original draft (equal); writing – review and editing (equal). **Jun Yao:** Investigation (equal); resources (equal). **Jianlin Long:** Investigation (equal); resources (equal). **Yong Mao:** Investigation (equal); resources (equal). **Di Wu:** Investigation (equal); resources (equal). **Aimin Zang:** Investigation (equal); resources (equal). **Jun Zhao:** Investigation (equal); resources (equal). **Ziling Liu:** Investigation (equal); resources (equal). **Rui Meng:** Investigation (equal); resources (equal). **Ye Chen:** Conceptualization (supporting); data curation (equal); formal analysis (equal); methodology (equal); writing – original draft (supporting); writing – review and editing (supporting). **Yang Luo:** Conceptualization (supporting); data curation (equal); formal analysis (equal); methodology (equal); writing – original draft (supporting); writing – review and editing (supporting). **Qun Guo:** Data curation (equal); formal analysis (equal); methodology (equal); visualization (supporting); writing – original draft (supporting); writing – review and editing (supporting). **Li Li:** Data curation (equal); formal analysis (equal); methodology (equal); visualization (supporting); writing – original draft (supporting); writing – review and editing (supporting). **Jiuwei Cui:** Conceptualization (lead); supervision (equal); validation (lead); writing – review and editing (lead).

## FUNDING INFORMATION

Funding for our study was provided by Innovent Biologics, Inc., Suzhou, Jiangsu, China. The authors and funder were involved in study design, collection, analysis, and interpretation of data and medical writing support. The funder maintained the study database.

## CONFLICT OF INTEREST STATEMENT

Yuguang Zhao, Xiao Chen, Jun Yao, Jianlin Long, Yong Mao, Di Wu, Aimin Zang, Jun Zhao, Ziling Liu, Rui Meng, Jiuwei Cui declared their institutions received study grants from Innovent Biologics, Inc., China. Ye Chen, Yang Luo, Qun Guo, Li Li, reported being employees of Innovent Biologics, Inc. Funding for the submitted work was as described above.

## ETHICS STATEMENT

The study protocol were approved by the appropriate institutional review boards and ethics review committees at each institution. The study was conducted in accordance with the protocol, Good Clinical Practice guidelines and the Declaration of Helsinki. All patients provided written informed consent for participating in the study.

## Supporting information


Data S1.

## Data Availability

The data that support the findings of our study are available from the corresponding author upon reasonable request.
