# Supplementary material for: A phase Ib study evaluating the safety and efficacy of IBI310 plus sintilimab in patients with advanced non‐small‐cell lung cancer who have progressed after anti‐PD‐1/L1 therapy
Source: Cancer Med. 2024 Jan 12;13(3):e6855. doi: 10.1002/cam4.6855 (PMC10905228; doi:10.1002/cam4.6855)
Supplement: Supplementary file 1 — Data S1. [file CAM4-13-e6855-s001.docx]

**Supplementary table 1. Treatment emergent adverse events in all patients**

|  | **cohort A:IBI310 1mg/kg**  **plus sintilimab**  **(n=15)** | | **cohort B: IBI310 3mg/kg plus sintilimab**  **(n=15)** | |
| --- | --- | --- | --- | --- |
|  | Any grade | ≥Grade 3 | Any grade | ≥Grade 3 |
| **Any** **treatment emergent adverse events^a^** | 15 (100) | 9 (60.0) | 14 (93.3) | 9 (60.0) |
| **Any-grade treatment emergent adverse events in 20% or more of treated patients in either group^b^** | | | | |
| Anaemia | 9 (60.0) | 1 (6.7) | 7 (46.7) | 1 (6.7) |
| Hypoalbuminaemia | 7 (46.7) | 0 | 9 (60.0) | 0 |
| Asthenia | 5 (33.3) | 0 | 6 (40.0) | 1 (6.7) |
| Rash | 5 (33.3) | 1 (6.7) | 2 (13.3) | 1 (6.7) |
| Alanine aminotransferase increased | 5 (33.3) | 1 (6.7) | 7 (46.7) | 0 |
| Aspartate aminotransferase increased | 4 (26.7) | 1 (6.7) | 6 (40.0) | 1 (6.7) |
| Blood alkaline phosphatase increased | 4 (26.7) | 1 (6.7) | 5 (33.3) | 0 |
| Weight decreased | 4 (26.7) | 0 | 0 | 0 |
| Decreased appetite | 4 (26.7) | 2 (13.3) | 5 (33.3) | 0 |
| Platelet count decreased | 3 (20.0) | 1 (6.7) | 5 (33.3) | 0 |
| Lipase increased | 3 (20.0) | 1 (6.7) | 4 (26.7) | 1 (6.7) |
| White blood cell count decreased | 3 (20.0) | 1 (6.7) | 2 (13.3) | 0 |
| Blood thyroid stimulating hormone increased | 3 (20.0) | 0 | 2 (13.3) | 0 |
| Hyponatraemia | 3 (20.0) | 0 | 4 (26.7) | 1 (6.7) |
| Hyperglycaemia | 3 (20.0) | 1 (6.7) | 4 (26.7) | 0 |
| Pyrexia | 3 (20.0) | 0 | 5 (33.3) | 0 |
| Cough | 3 (20.0) | 0 | 0 | 0 |
| C-reactive protein increased | 2 (13.3) | 0 | 6 (40.0) | 1 (6.7) |
| Gamma-glutamyltransferase increased | 2 (13.3) | 0 | 4 (26.7) | 2 (13.3) |
| Protein urine present | 2 (13.3) | 0 | 4 (26.7) | 0 |
| Blood lactate dehydrogenase increased | 2 (13.3) | 1 (6.7) | 3 (20.0) | 0 |
| Hypocalcaemia | 2 (13.3) | 0 | 3 (20.0) | 0 |
| Pneumonia | 2 (13.3) | 0 | 3 (20.0) | 0 |
| Occult blood positive | 1 ( 6.7) | 0 | 3 (20.0) | 0 |
| Blood urea increased | 1 (6.7) | 0 | 3 (20.0) | 0 |
| Blood bilirubin increased | 1 (6.7) | 0 | 3 (20.0) | 0 |
| Hypokalaemia | 0 | 0 | 3 (20.0) | 0 |
| Hypomagnesaemia | 0 | 0 | 3 (20.0) | 0 |
| Hepatic function abnormal | 0 | 0 | 4 (26.7) | 1 (6.7) |

Data are n (%).

^a^ Adverse events were classified according to *Medical Dictionary for Regulatory Activities* and graded according to the National Cancer Institute Common Terminology Criteria for Adverse Events, version 5.0. Grading ranges from 1 through 5 (1, mild; 2, moderate; 3, severe; 4, life-threatening; and 5, death).

**Supplementary table 2. Summary of immune-related adverse events with systemic corticosteroids**

|  | **cohort A:IBI310 1mg/kg**  **plus sintilimab**  **(n=15)** | | **cohort B: IBI310 3mg/kg plus sintilimab**  **(n=15)** | | **All patients** | |
| --- | --- | --- | --- | --- | --- | --- |
|  | Any grade | ≥Grade 3 | Any grade | ≥Grade 3 | Any grade | ≥Grade 3 |
| Any immune-related adverse events | 7(46.7) | 3(20.0) | 7(46.7) | 4(26.7) | 14(46.7) | 7(23.3) |
| **Immune-related adverse events in 10% or more of patients in either group**^a^ | | | | | | |
| Alanine aminotransferase increased | 2(13.3) | 1(6.7) | 0 | 0 | 2(6.7) | 1( 3.3) |
| Aspartate aminotransferase increased | 2(13.3) | 0 | 0 | 0 | 2(6.7) | 0 |
| Adrenal insufficiency | 2(13.3) | 0 | 0 | 0 | 2(6.7) | 0 |
| Rash | 2(13.3) | 1(6.7) | 2(13.3) | 1(6.7) | 4(13.3) | 2(6.7) |
| Hepatic function abnormal | 0 | 0 | 3(20.0) | 1(6.7) | 3(10.0) | 1(3.3) |

Data are n (%).

^a^ Immune-related adverse events assessed by investigators.

**Supplementary table 3. Safety Summary**

|  | **cohort A:IBI310 1mg/kg**  **plus sintilimab**  **(n=15)** | **cohort B: IBI310 3mg/kg plus sintilimab**  **(n=15)** | **All patients**  **(n=30)** |
| --- | --- | --- | --- |
| All Grade TRAEs, n (%) | 13 (86.7) | 13 (86.7) | 26 (86.7) |
| Grade ≥ 3 TRAEs, n (%) | 6 (40.0) | 8 (53.3) | 14 (46.7) |
| TRAE leading to drug interruption, n (%) | 6 (40.0) | 7 (46.7) | 13 (43.3) |
| TRAE leading to drug discontinuation, n (%) | 1 (6.7) | 0 | 1 (3.3) |
| TRAE leading to death, n (%) | 1 (6.7) | 2 (13.3) | 3 (10.0) |
| IrAE leading to drug discontinuation^a^, n (%) | 1 (6.7) | 0 | 1 (3.3) |

Data are n (%). Treatment-related adverse event was related to any study drug.

Adverse events were classified according to *Medical Dictionary for Regulatory Activities* and graded according to the National Cancer Institute Common Terminology Criteria for Adverse Events, version 5.0. Grading ranges from 1 through 5 (1, mild; 2, moderate; 3, severe; 4, life-threatening; and 5, death).

^a^ Immune-related adverse events assessed by investigators.
